# Conservative Management of a Primary Mandibular Second Molar With Dentoalveolar Abscess in a Four-Year-Old Child With Acute Lymphoblastic Leukemia: A Case Report

**DOI:** 10.7759/cureus.114469

**Published:** 2026-08-13

**Authors:** Aswathy Sudarsanan, Elaine S Barretto, Dinesh F Swamy, Ruhi Sanvordekar, Devika Sajeev

**Affiliations:** 1 Department of Pediatric and Preventive Dentistry, Goa Dental College and Hospital, Bambolim, IND

**Keywords:** acute lymphoblastic leukemia (all), clinical pediatric dentistry, dentoalveolar abscess, oral rehabilitation, pulpectomy, stainless steel crown

## Abstract

Acute lymphoblastic leukemia (ALL) is the most common pediatric hematologic malignancy and is frequently associated with significant oral complications resulting from the disease as well as the treatment-related immunosuppression. In such conditions, odontogenic infections may progress rapidly and contribute to systemic morbidity, thereby necessitating proper and carefully coordinated dental management. A four-year-old boy diagnosed with ALL, in the maintenance phase of chemotherapy, presented with pain and swelling in the lower right back tooth region for five days. Clinical and radiographic examination revealed a dentoalveolar abscess associated with the right deciduous mandibular second molar, along with multiple carious lesions involving other teeth. Following consultation with the pediatric oncologist and assessment of hematological parameters, a comprehensive dental rehabilitation was performed under strict infection control measures. Treatment included a pulpectomy of the right deciduous mandibular second molar, followed by placement of a stainless-steel crown and restorative management of other carious lesions. The child demonstrated complete resolution of pain and swelling, preservation of the affected primary tooth, restoration of masticatory function, and significant improvement in oral health during the follow-up visits. No postoperative complications or interruption of ongoing chemotherapy occurred. This case highlights that conservative management of an infected primary molar can be considered and safely done in children with ALL if their medical condition and hematological status permit. Also, careful treatment planning and multidisciplinary coordination with the oncologist allowed elimination of the oral foci of infection while preserving the teeth and maintaining arch integrity.

## Introduction

Acute lymphoblastic leukemia (ALL) is the most common childhood malignancy, accounting for nearly one-third of cancers diagnosed in children, and remains the predominant hematological cancer affecting individuals younger than 15 years of age [1,2]. Advances in chemotherapy have markedly improved survival outcomes over the past few decades. Nevertheless, the oral health needs of these children continue to present a significant clinical challenge, largely because of treatment-related immunosuppression, thrombocytopenia, and the increased susceptibility to infection that accompanies intensive cancer therapy [2, 3].

The oral cavity frequently reflects both the direct manifestations of leukemia and the adverse effects of antineoplastic therapy. Common oral findings include gingival inflammation, mucositis, spontaneous bleeding, mucosal pallor, xerostomia, opportunistic infections, delayed wound healing, and extensive dental caries [2-4]. Odontogenic infections in pediatric leukemia patients are of particular concern, as they can act as persistent sources of microbial challenge in immunocompromised patients and, if left untreated, have the potential to interfere with ongoing medical management [3].

Current pediatric oncology guidelines emphasize the elimination of all active and potential oral infectious foci prior to and during chemotherapy whenever possible [3,5]. Dental management in such medically compromised children requires careful evaluation of hematological parameters, treatment timing, and communication with the oncologist to minimize the risks and complications.

The present case report describes the management of a dentoalveolar abscess associated with a primary mandibular second molar in a four-year-old child undergoing maintenance chemotherapy for ALL. The clinical considerations that influenced the decision to preserve the affected tooth through pulpectomy and stainless-steel crown rehabilitation, rather than extraction, are discussed.

## Case presentation

A four-year-old boy was referred from the Department of Pediatrics, Goa Medical College, Bambolim, to the Department of Pediatric and Preventive Dentistry, Goa Dental College and Hospital, Bambolim, India, with a chief complaint of pain and swelling in the lower right back tooth region for five days.

The child had been diagnosed with ALL two years earlier and was undergoing maintenance-phase chemotherapy under regular oncologic supervision. The medical history revealed no known drug allergies or previous adverse reactions to dental treatment. Hematological investigations demonstrated values within acceptable limits for dental treatment (hemoglobin: 11.5 g/dL, platelet count: 232 x 10³/uL, absolute neutrophil count: 1.64 x 10³/uL).

Prior to dental intervention, pediatric oncologist consultation was done, and clearance for dental treatments was obtained.

Extraoral examination revealed mild diffuse swelling over the right mandibular region with localized tenderness on palpation and enlarged submandibular lymph nodes. No significant facial asymmetry was observed.

**Figure 1:**
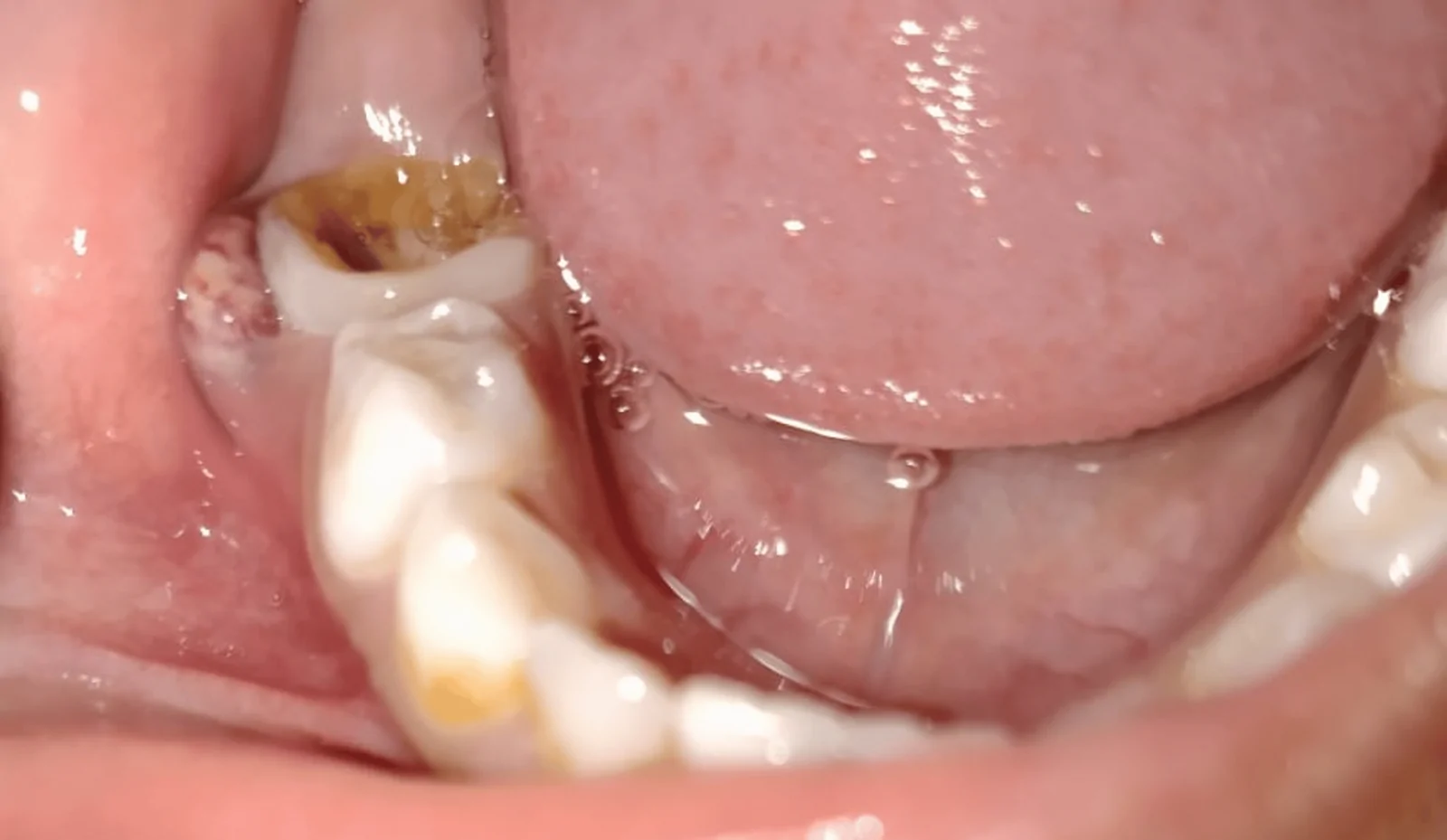
Preoperative intraoral photograph showing the carious primary right mandibular second molar associated with a dentoalveolar abscess.

**Figure 2:**
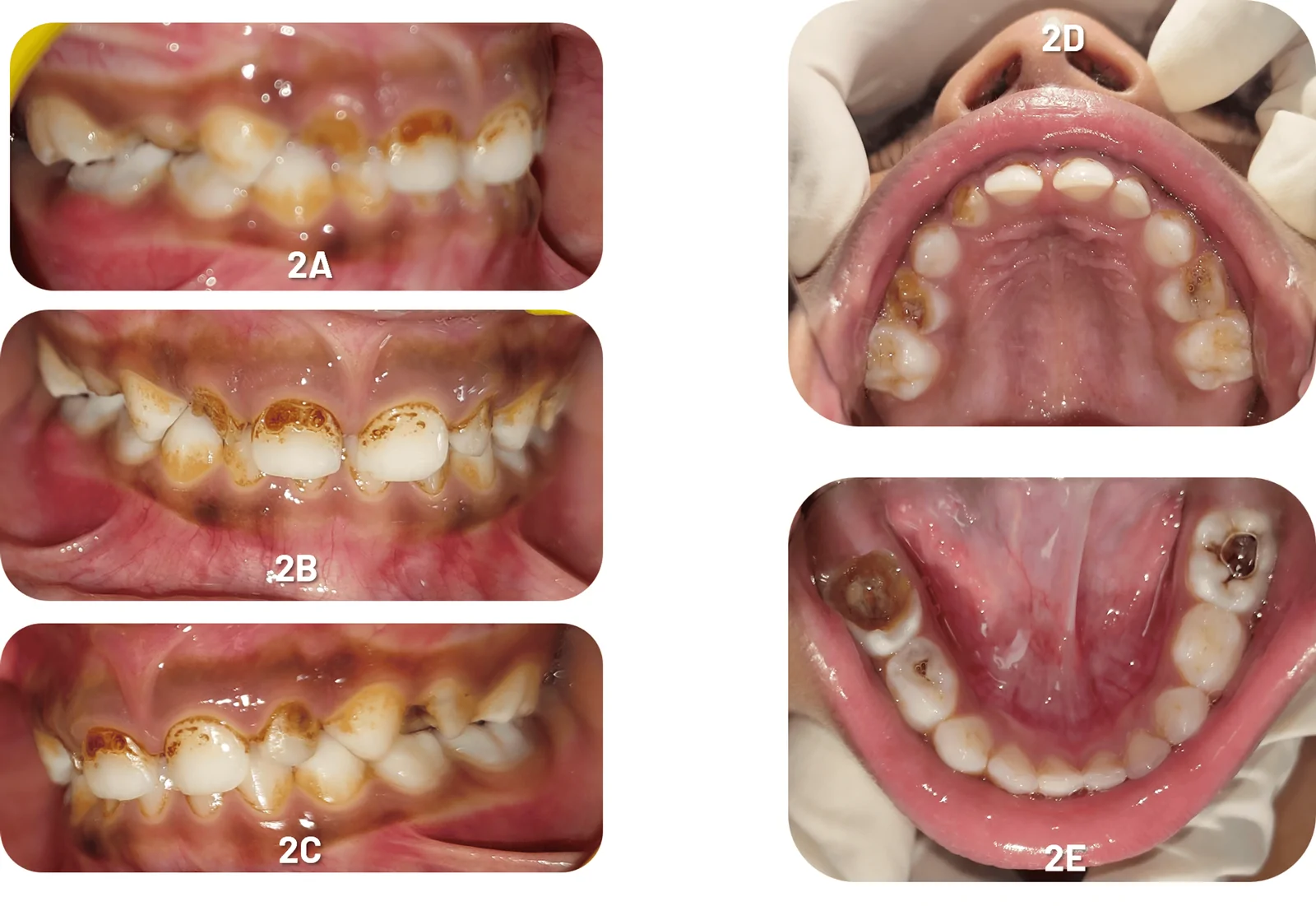
Preoperative intraoral photographs 2A: right lateral view; 2B: frontal view; 2C: left lateral view; 2D: maxillary occlusal view; 2E: mandibular occlusal view

Intraoral examination revealed extensive caries with a dentoalveolar abscess on the right deciduous mandibular second molar as shown in Figure 1. Also, multiple other carious teeth were present. Figure 2 shows the preoperative intraoral photographs.

After taking informed consent from the parent, diet analysis and counseling were done. After consultation with the oncologist, antibiotics were prescribed to manage the dentoalveolar infection, along with analgesics for pain control, prior to definitive dental treatment. Local anesthesia was given. Following caries excavation and access opening on the right mandibular second molar, necrotic pulpal tissue was extirpated. Biomechanical preparation was performed using hand files, with copious irrigation using 0.9% normal saline. The canals were subsequently dried using paper points and obturated using Metapex (Meta Biomed Co., Ltd., Cheongju-si, Chungbuk, Republic of Korea). A glass ionomer cement base was placed to achieve coronal seal integrity.

Following pulpectomy, tooth preparation for stainless steel crown placement was done on the right mandibular second molar. Appropriate crown size selection, adaptation, contouring, and crimping were performed to achieve optimal marginal fit and occlusal harmony. Then the crown was cemented on the right mandibular second molar using luting glass ionomer cement. 

**Figure 3:**
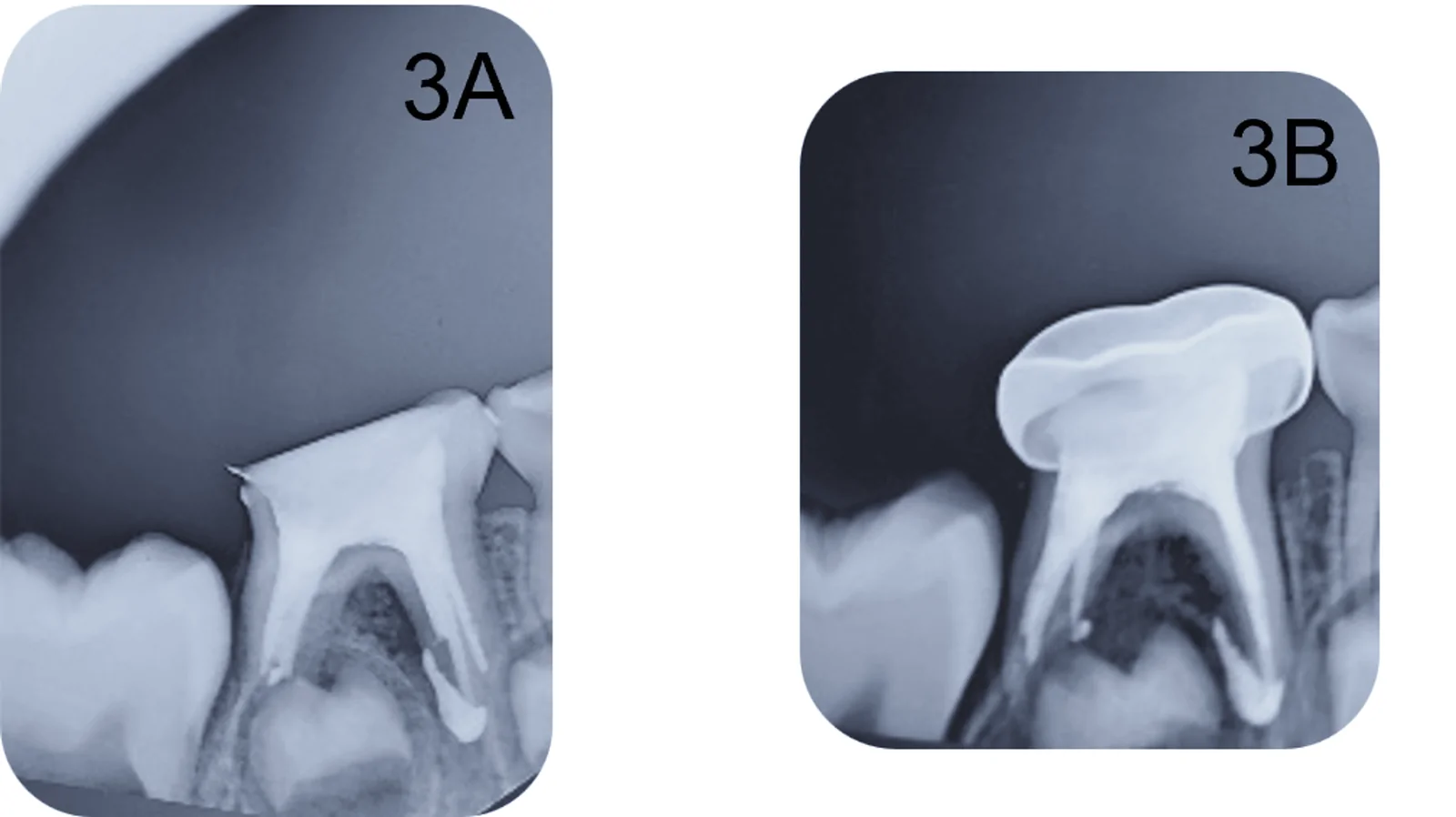
Postoperative radiographs obtained after pulpectomy (A) and placement of a stainless-steel crown on the right mandibular second molar (B).

Figures 3A, 3B show postoperative radiographs after pulpectomy of the right mandibular second molar and placement of a stainless steel crown on the right mandibular second molar, respectively. All the other carious lesions were restored using restorative glass ionomer cement and composite restorations.

Postoperative instructions were explained to the parents, and regular follow-up visits were scheduled.

At one-month follow-up, complete resolution of pain and swelling was observed. Soft tissue healing was satisfactory, and there was no evidence of recurrent infections.

**Figure 4:**
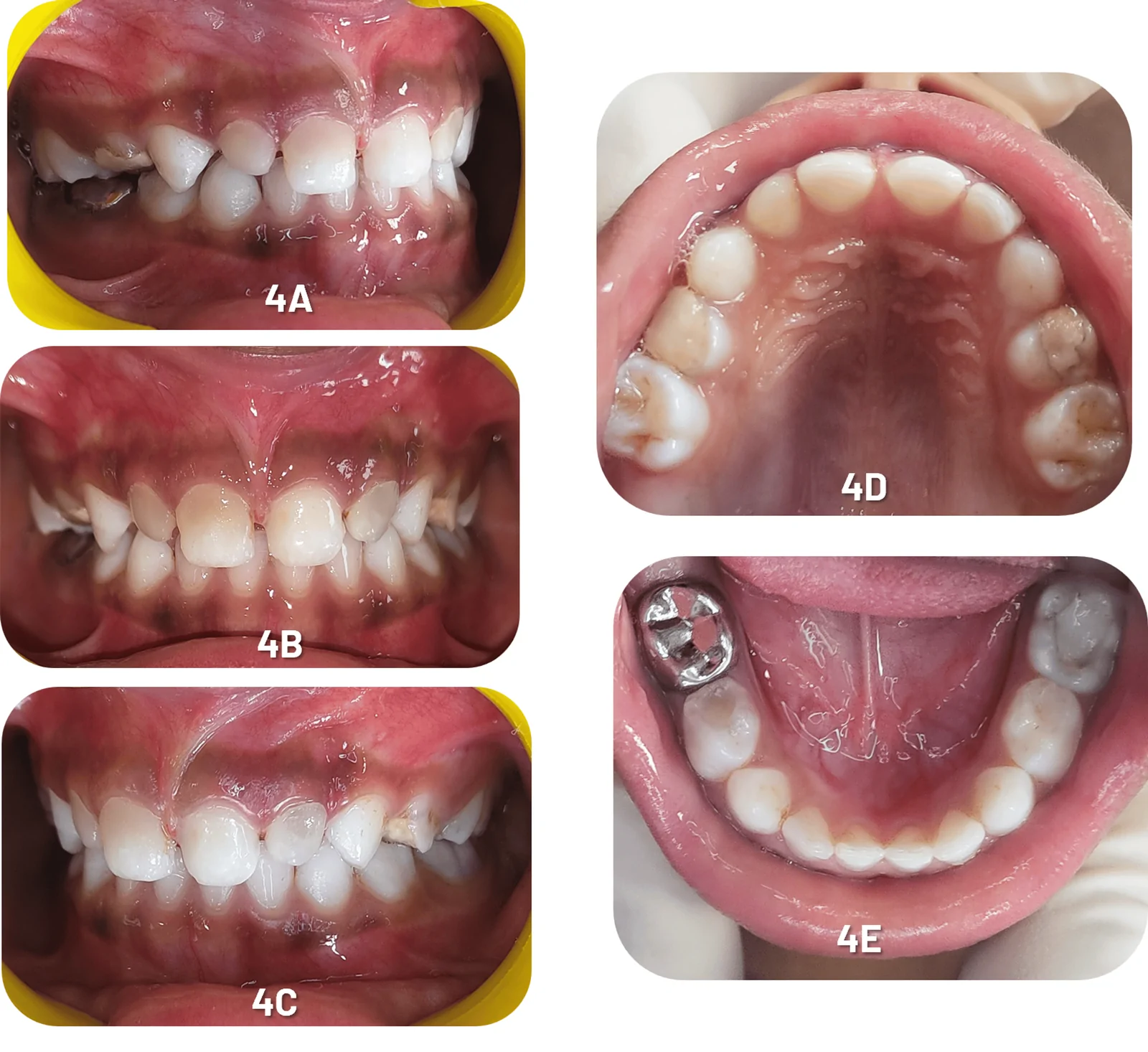
Six-month postoperative follow-up intraoral photographs. 4A: right lateral view; 4B: frontal view; 4C: left lateral view; 4D: maxillary occlusal view; 4E: mandibular occlusal view

During the three-month, six-month (Figure 4), and one-year recall visits, the right mandibular second molar pulpectomized teeth remained asymptomatic and functional, and the stainless-steel crown demonstrated satisfactory retention. Oral hygiene of the child improved significantly, and there was no interruption of chemotherapy that occurred secondary to dental foci of infection.

## Discussion

Children undergoing chemotherapy for ALL are particularly susceptible to oral disease due to immunosuppression, altered salivary function, poor oral hygiene maintenance, and dietary modifications associated with cancer therapy [2-4]. Odontogenic infections in these patients may progress rapidly and represent a potential source of life-threatening systemic infection [3].

The present case highlights the role of prompt dental intervention and careful medical consultation in minimizing potential complications associated with odontogenic infections in children with leukemia. Consultation with the pediatric oncologist and evaluation of hematological parameters prior to treatment were essential to minimize the risk of bleeding and infection-related complications.

Maintenance phase chemotherapy generally permits safer delivery of dental care compared with induction phases, provided hematological indices are within acceptable limits [3]. According to the American Academy of Pediatric Dentistry guidelines, dental treatment may be safely performed in children with adequate platelet and neutrophil counts following medical clearance [5].

Although the American Academy of Pediatric Dentistry lists pulp therapy as a relative contraindication in children with malignancies [5], the treatment plan in this case was guided by the patient's overall medical condition and hematological status. The child was in the maintenance phase of chemotherapy and had demonstrated consistently stable blood parameters, which supported consideration of a conservative approach. Extraction of the right mandibular second molar was carefully evaluated but ultimately avoided because the permanent mandibular first molar had not yet erupted. Premature loss of the second primary molar would have necessitated the use of a distal shoe space maintainer to maintain arch integrity. As the placement of such appliances may be associated with additional concerns in children with hematological disorders, including the potential for soft-tissue irritation and increased risk of infection, retaining the tooth was considered the more favorable option. Therefore, pulpectomy followed by stainless steel crown restoration on the right mandibular second molar was carried out to eliminate infection while preserving function and arch length.

The successful outcome observed in this case emphasizes that comprehensive dental rehabilitation can be safely accomplished in pediatric leukemia patients when evidence-based protocols and multidisciplinary coordination are followed. Also, as this report describes a single case, the findings cannot be generalized, and longer-term follow-up is needed to confirm the sustained treatment outcome.

## Conclusions

The management of odontogenic infections in children undergoing treatment for ALL presents unique clinical challenges, often requiring modification of conventional treatment approaches. In the present case, successful resolution of the dentoalveolar abscess was achieved through conservative rehabilitation of a strategically important primary second molar following careful evaluation of the patient's medical status and hematological stability. Retaining the tooth preserved arch integrity and eliminated the need for additional interventions that could further complicate care in an immunocompromised child. This case illustrates that treatment decisions in pediatric oncology patients should be guided not only by the presence of oral pathology but also by an assessment of future developmental and functional implications. When undertaken in consultation with the oncology team, conservative dental management may represent a predictable and clinically sound alternative to extraction in selected cases.
